# *Leishmania tropica* Infection in Golden Jackals and Red Foxes, Israel

**DOI:** 10.3201/eid1612.100953

**Published:** 2010-12

**Authors:** Dalit Talmi-Frank, Noa Kedem-Vaanunu, Roni King, Gila Kahila Bar-Gal, Nir Edery, Charles L. Jaffe, Gad Baneth

**Affiliations:** Author affiliations: Hebrew University, Rehovot, Israel (D. Talmi-Frank, N. Kedem-Vaanunu, G. Kahila Bar-Gal, G. Baneth);; Israel Nature and Parks Authority, Jerusalem, Israel (R. King);; Kimron Veterinary Institute, Bet-Dagan, Israel (N. Edery);; Hebrew University–Hadassah Medical School, Jerusalem (C.L. Jaffe)

**Keywords:** Leishmania tropica, red fox, golden jackal, Israel, zoonoses, bacteria, dispatch

## Abstract

During a survey of wild canids, internal transcribed spacer 1 real-time PCR and high-resolution melt analysis identified *Leishmania tropica* in samples from jackals and foxes. Infection was most prevalent in ear and spleen samples. Jackals and foxes may play a role in the spread of zoonotic *L. tropica*.

*Leishmania tropica* is a major cause of cutaneous leishmaniasis in the Old World. Although cutaneous leishmaniasis associated with *L. tropica* usually is considered an anthroponotic infection (*1*) in Israel, Jordan, and the Palestinian Authority, it appears to be a zoonosis with a main putative reservoir host, the rock hyrax (*Procavia capensis*) (*2**,**3*). Nevertheless, the possible involvement of other animals in the sylvatic transmission of *L. tropica* infection is not yet fully understood. *L. tropica* has been sporadically reported from domestic dogs from human cutaneous leishmaniasis foci in Iran and Morocco (*4**,**5*) but not from wild canids. Previous studies of leishmaniasis in wild canids, such as red foxes (*Vulpes vulpes*) in southern Italy (*6*) and wolves (*Canis lupus*) in southwestern Europe (*7*), found them to be infected with *L. infantum.* Golden jackals (*Canis aureus*) infected with *L. infantum* were reported in Iraq (*8*) and Kazakhstan (*9*). A seroepidemiologic study of *Leishmania* spp. infection in Israel showed that 7.6% of jackals and 5% of foxes tested were seropositive by using *L. donovani* antigen (*10*). The aim of this study was to identify and characterize *Leishmania* spp. infection in wild canids, including jackals, foxes, and wolves, in Israel by using species-specific real-time PCR.

## The Study

Wild golden jackals, red foxes, and gray wolves were trapped at 57 different locations in Israel as part of a survey for oral rabies vaccination conducted by the Israeli Nature and Parks Authority and the Veterinary Services. DNA was extracted from ear pinna, snout, blood, and spleen by using the guanidine thiocyanate technique (*11*); in some animals, samples were not available from all sites. DNA from all tissues was tested for *Leishmania* spp. infection by internal transcribed spacer 1 (ITS1) real-time PCR and high-resolution melt analysis (ITS1-HRM) PCR (*12*). A 265–288-bp fragment, depending on the *Leishmania* species, within the ITS1 region of the leishmanial rRNA was amplified as previously described (*12*). All samples were tested in duplicates and results were compared with those from HRM analysis of positive controls for each assay. These were *L. infantum* (MCAN/IL/2002/Skoshi), *L. tropica* (MHOM/IL/2005/LRC-L1239), and *L. major* (MHOM/TM/1973/5ASKH). Negative controls included samples from jackals born and reared at a zoo in central Israel, as well as from foxes and wolves from areas in which leishmaniasis is not endemic, that were tested by PCR and found negative. All positive PCR products were purified by using ExoSAP-IT (USB, Cleveland, OH, USA) and sequenced at the Center for Genomic Technologies, Hebrew University of Jerusalem. Sequences obtained were compared for similarity to sequences in GenBank by using the BLAST program (www.ncbi.nlm.nih.gov/BLAST). Positive samples also were verified by kDNA PCR as described (*13*).

Sequences were analyzed by using MEGA version 3.0 (www.megasoftware.net). A phylogenetic tree was constructed by using the neighbor-joining method in agreement with maximum-parsimony and maximum-evolution algorithms and by using the Kimura 2-parameter model with uniform rates for transitions and transversions. Bootstrap replicates were performed to estimate the node reliability, and values were obtained from 1,000 randomly selected samples of the aligned sequence data. Sequences were compared with the following *Leishmania* sequences deposited in GenBank: *L. tropica* FJ595949 and FJ595950 from central Israel and IARA/IL/02/LRC-L910 and ISER/IL/02/LRC-L909 from northern Israel; *L. infantum* (MHOM/TN/1980/IPT1) and *L. major* (MHOM/TM/1973/5ASKH) were used as outgroups.

We examined 208 samples from 113 wild canids by ITS1-HRM PCR: 152 samples from 77 golden jackals, 44 from 25 red foxes, and 12 samples from 11 wolves. None of the animals had clinical signs attributed to leishmaniasis. Seven animals tested positive for *L. tropica*, and 1 was positive for *L. infantum*. The overall *Leishmania* infection rate for jackals was 7.8% (6/77) and for foxes 8% (2/25). All wolves were negative. Fourteen tissue samples (ear, snout, spleen, and blood) tested positive. Five (63%) of the 8 animals positive for *Leishmania* spp. had >2 infected tissues (Table). Ears were positive for 6 of 8 infected animals and spleen for 4 of 8 animals. The snout sample was positive for another animal for which blood also was positive. Four (15%) of 26 spleens collected were positive.

**Table:** Sequence similarity obtained for *Leishmania* spp. ITS1-positive tissue samples from jackals and foxes, Israel*

Subject no.	Animal species	Tissue	HRM results	ITS1 sequence length (% similarity†)	GenBank accession nos.	
					Comparison isolate	Identified isolate
918	Jackal	Right ear, left ear	*L. tropica*	236–239 bp (98)	FJ948456	GU591390
922	Jackal	Right ear, left ear	*L. tropica*	235–239 bp (98)	FJ948456	GU591391
1067	Jackal	Right ear, spleen	*L. tropica*	233–237 bp (98)	FJ948456	GU591392
1086	Jackal	Right ear, spleen	*L. tropica*	234–238 bp (98)	FJ948456	GU591393
1380	Jackal	Spleen	*L. tropica*	235–239 bp (98)	FJ948456	GU591394
115	Jackal	Blood	*L. infantum*	221–222 bp (99)	GU045592	GU591395
1084	Fox	Right ear, snout, spleen	*L. tropica*	234–239 bp (98)	FJ948456	GU591396
579916	Fox	Left ear	*L. tropica*	235–239 bp (98)	FJ9484556	GU591397

*ITS1, internal transcribed spacer 1; HRM, high-resolution melt analysis. †Similarity to comparison isolate by BLAST (www.ncbi.nlm.nih.gov/BLAST).

The ITS1-HRM PCR DNA product size was 265 bp for *L. infantum* and 273 bp for *L. tropica*. Sequencing verified the species specific results. All samples positive by ITS1-HRM PCR were also positive by kDNA PCR and produced a 120-bp kDNA product.

Thirteen sequences from positive DNA products obtained by ITS1-HRM PCR were identified as belonging to *L. tropica,* showing the closest similarity (98%–99%) to *L. tropica* sequences deposited in GenBank (Table). Only 1 sequence was amplified by using DNA extracted from the blood of a jackal for which other tissues were not available. This sequence was closest (99.5% identity over 222 bp) to *L. infantum* (Table). DNA sequences from all the positive tissues belonging to an individual animal were aligned, and consensus sequences representing each animal were created. These consensus sequences were deposited in GenBank under accession nos. GU591390–GU591397 and included in the phylogenetic tree (Figure). By using 3 algorithms, sequences obtained from 7 wild canids clustered with *L. tropica* isolated from hyraxes in central Israel (FJ595949 and FJ595950), and the sample amplified from the jackal blood clustered with *L. infantum* (MHOM/TN/1980/IPT1).

**Figure:**
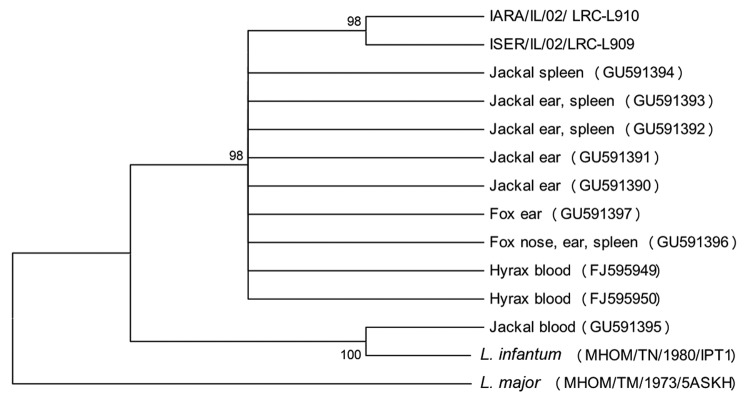
Neighbor-joining tree phylogram comparing internal transcribed spacer 1 (ITS1) *Leishmania tropica* DNA sequences from wild canids, Israel. The neighbor-joining tree constructed in MEGA version 3.0 (www.megasoftware.net) by the ITS1 HRM PCR sequences (222–239 nt) agrees with the maximum-likelihood algorithm. The tree shown is based on the Kimura 2-parameter model of nucleotide substitution. Bootstrap values are based on 1,000 replicates. The analysis provided tree topology only; the lengths of the vertical and horizontal lines are not significant. *L. major* was used as an outgroup. GenBank accession numbers of *L. tropica* from hyraxes deposited from this study are shown in brackets. Numbers on nodes represent bootstrap values. MHOM, human; IARA, *Phlebotomus arabicus* sand fly; ISER, *Phlebotomus sergenti* sandfly.

## Conclusions

We report *L. tropica* infections in jackals and foxes from Israel. Sequence analysis (using 3 algorithms) of ITS1 fragments showed perfect correlation with *L. tropica* isolates from hyraxes in central Israel. The finding of *L. tropica* positivity in >1 tissue sample from infected asymptomatic animals implies that wild canid species could be natural hosts for this parasite. Furthermore, the relatively high percentage of infected spleens indicates that this parasite can visceralize in foxes and jackals. Unlike hyraxes, which generally stay close to their burrows in caves or boulders, jackals and foxes travel long distances, potentially transmitting *L. tropica* from 1 area to another, provided that competent sandfly vectors are found. The home range of golden jackals in Israel is adapted to the food resources available. For golden jackals, it was 6.6 km^2^ near settlements and 21.2 km^2^ in sparsely inhabited settings (*14*). Foxes may foray 5.3 km and less frequently roam 7.8 km–15 km (*15*). Wild canids may transmit *L. tropica* from an area with an infected population of hyraxes to a remote naive hyrax population or be responsible for infecting humans because they tend to live peridomestically and frequently rely on human waste. The involvement of wild canids in the sylvatic life cycle of *L. tropica* could be crucial to understanding disease emergence in Israel, Jordan, and the Palestinian Authority (*2*). Populations of jackals and foxes, which were nearly eliminated in Israel during 1950–1980, have recovered and grown in parallel with the local outbreaks of *L. tropica* in humans (*10*). Further study is required to discern the potential epidemiologic role of wild canids in spreading and transmitting infection.
